# Recent Advances in Microfluidic Platform for Physical and Immunological Detection and Capture of Circulating Tumor Cells

**DOI:** 10.3390/bios12040220

**Published:** 2022-04-07

**Authors:** Mahesh Padmalaya Bhat, Venkatachalam Thendral, Uluvangada Thammaiah Uthappa, Kyeong-Hwan Lee, Madhuprasad Kigga, Tariq Altalhi, Mahaveer D. Kurkuri, Krishna Kant

**Affiliations:** 1 Centre for Research in Functional Materials (CRFM), Jain Global Campus, Jain University, Bengaluru 562112, Karnataka, India; maheshbhat1306@gmail.com (M.P.B.); thendralsasivenkat@gmail.com (V.T.); madhuprasad@jainuniversity.ac.in (M.K.); 2 Agricultural Automation Research Center, Chonnam National University, Gwangju 61186, Korea; khlee@chonnam.ac.kr; 3 School of Chemical Engineering, Yeungnam University, 280 Daehak-ro, Gyeongsan 38541, Korea; sanjuuthappa@gmail.com; 4 Department of Convergence Biosystems Engineering, Chonnam National University, Gwangju 61186, Korea; 5 Department of Chemistry, Faculty of Science, Taif University, Taif 21944, Saudi Arabia; tmmba@windowslive.com; 6 Departamento de Química Física, Campus Universitario, CINBIO Universidade de Vigo, 36310 Vigo, Spain

**Keywords:** circulating tumor cells (CTCs), microfluidic device, physical method, biological method, cancer diagnostics

## Abstract

CTCs (circulating tumor cells) are well-known for their use in clinical trials for tumor diagnosis. Capturing and isolating these CTCs from whole blood samples has enormous benefits in cancer diagnosis and treatment. In general, various approaches are being used to separate malignant cells, including immunomagnets, macroscale filters, centrifuges, dielectrophoresis, and immunological approaches. These procedures, on the other hand, are time-consuming and necessitate multiple high-level operational protocols. In addition, considering their low efficiency and throughput, the processes of capturing and isolating CTCs face tremendous challenges. Meanwhile, recent advances in microfluidic devices promise unprecedented advantages for capturing and isolating CTCs with greater efficiency, sensitivity, selectivity and accuracy. In this regard, this review article focuses primarily on the various fabrication methodologies involved in microfluidic devices and techniques specifically used to capture and isolate CTCs using various physical and biological methods as well as their conceptual ideas, advantages and disadvantages.

## 1. Introduction

Cancer is defined as the uncontrolled proliferation of aberrant cells in the human body, and it is classified into two types: benign and malignant cancers. A benign tumor that grows slowly and has no negative effects on the human body. Malignant tumors, on the other hand, are aggressive, grow quickly, spread rapidly and eventually kill the patient. During metastasis, some tumor cells at the primary tumor’s borders undergo a process known as epithelial-mesenchymal transition (EMT), in which the cells lose their epithelial traits and gain migratory mesenchyme properties [1]. These migratory tumor cells enter adjacent arteries and start travelling along with red and white blood cells throughout the body. CTCs enter the bloodstream through the vasculature and circulate alongside healthy hematological cells before metastasis [2,3]. However, these can only be diagnosed if the patient has progressed to the metastatic stage [4]. These CTCs stop internally at some organs and trigger secondary tumors; from this stage onwards, the cancer enters its deadliest form, and the patient could face fatal consequences [5,6]. Hence, the early detection of these cells or the monitoring of their presence in the bloodstream is required and important for the accurate diagnosis and prognosis of cancer [7]. A survey has shown that malignant tumors will be the major cause of death worldwide by 2030, expected to grow to 20.3 million new cancer cases and 13.2 million deaths [8]. 

However, CTCs are extremely rare among hematological cells. There are only a few CTCs in a 1.0 mL blood sample, where nearly 5 billion red blood cells (RBCs) and 10 million white blood cells (WBCs) are present. In addition, the CTCs may exist in a single-cell or cluster form, with varied phenotypic properties. Based on the changes in protein expression on CTCs, they can be classified into epithelial-mesenchymal, epithelial, and mesenchymal types [9]. Therefore, collecting and isolating them from other components in the bloodstream is quite difficult and challenging [10]. Detection of these rare cells using sensors would be beneficial. Sensors have previously been used for environmental applications [11,12,13,14,15,16,17]. On the other hand, sensors would be ideal for the detection of these rare CTCs. Currently, several techniques such as flow cytometry, enzyme-linked immunosorbent assay (ELISA), Western blotting, quantitative polymerase chain reaction (Q-PCR), magnetic-activated cell sorting (MACS), fluorescence-activated cell sorting (FACS) and centrifugation techniques, and laser-based technology are widely used for the biomolecular or cellular analysis of cancer [18,19,20,21,22,23,24,25,26]. Although these techniques have several limitations, such as substantial sample consumption, low throughput, lack of real-time monitoring, and high overall operational expenses, there are no other alternative simple techniques available for CTC isolation. As a result, there is a great scientific desire to improve cancer diagnosis using low-cost procedures [27]. 

**Figure 1:**
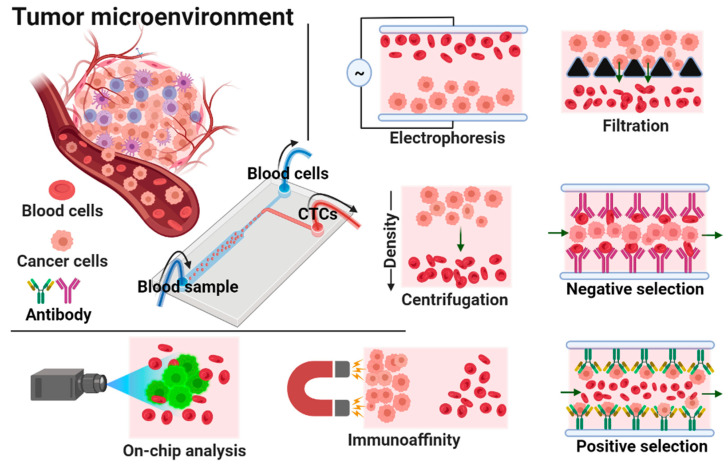
Range of methods involved in physical and biological approaches for early-stage detection and isolation of CTCs.

In our opinion, microfluidic devices are one of the most intriguing methods for capturing and isolating CTCs from blood samples. Microfluidic devices have many advantages, including their high throughput, low cost, miniaturization, quick analysis, high sensitivity, precise operation, high efficiency, portability, low sample consumption, and accuracy [28,29,30,31,32,33,34]. As the name implies, microfluidics is concerned with accurate fluid flow management in microliters (10–6) to picoliters (10–12) within micro-volume channels [35]. Various techniques like 3D printing [36], molding, laminating, and high-resolution nanofabrication are used to create these devices. S.C. Terry reported the first lab-on-a-chip (LOC) analysis system in 1979, which was investigated for gas chromatography applications [37]. Since then, microfluidic devices have been investigated for a variety of applications, including biosensors [38], separation [39], analysis [40], drug delivery [41,42], optoelectronics [43], cell manipulation [44], and chemical synthesis [45,46]. There has been much advancement in surface chemistry, which has enabled the development of smart surfaces and devices for various applications [47,48]. In comparison to other approaches, microfluidic channels have a high surface-to-volume ratio [49]. Microfluidic devices are usually made from polymers such polyethylene glycol diacrylate (PEGDA) [50], parylene [51], and polydimethylsiloxane (PDMS) [52,53]. There are two types of microfluidic technologies for capturing and isolating CTCs: physical and biological methods [54]. The different intrinsic features of cell populations, including their density [55], size [56], compressibility [57], deformability [58], dielectric properties [59], and viscosity [60], are used to physically separate CTCs [61,62]. Deterministic lateral displacement, inertial microfluidics, micropores, micropillar arrays, vortex-mediated deformability cytometry (VDC), inertial focusing dielectrophoresis, acoustic waves, and optical approaches have all been reported for the detection and separation of CTCs [63,64,65,66,67,68,69]. Biological approaches, on the other hand, rely on specific surface proteins produced on tumor cells to act as molecular recognizers such as transferrin, peptides, sialic acid, and antibodies to trap and isolate CTCs [70,71,72]. The two primary kinds of biological techniques are positive and negative sorting. The epithelial cell adhesion molecule (EpCAM) is a one-of-a-kind biomarker for positive sorting, which uses CTCs as target cells. Negative sorting, on the other hand, uses CD1513, anti-CD6647, and anti-CD45 biomarkers to identify leukocytes as target cells. Physical approaches are simple to use and do not require expensive biomarkers or a long incubation period. Nonetheless, they lack specificity and isolation purity. Biological approaches, on the other hand, need a more involved, time-consuming, and costly procedure. Yet, they have a high level of specificity, purity, and efficiency [73]. As a result, a physical-based approach is one of the most effective and straightforward methods for capturing and isolating CTCs. In this review, we have highlighted and critically examined the recent relevant literature on the fabrication of microfluidic devices for CTC isolation and the most promising elements of CTC capture and isolation, employing innovative microfluidic devices such as physical and biological techniques. The general technologies involved in the physical and biological separation of CTCs are depicted in Figure 1.

## 2. Fabrication of Microfluidic Devices for the Isolation of CTCs

In a very short span of time, microfluidics has emerged in several technological advancements. There are a variety of materials for microfluidic device fabrication, each with different properties according to the requirements. Based on the required specific characteristics of the fabrication material and product requirements, different techniques are used for the development of the device. Another major aspect is the cost of the involved material. In most cases, used devices are disposed of. Thus, the method involved should be economically feasible. Herein, we have classified the most recent techniques adapted in the fabrication of microfluidic devices for the isolation of CTCs.

### 2.1. Additive Manufacturing

**Figure 2:**
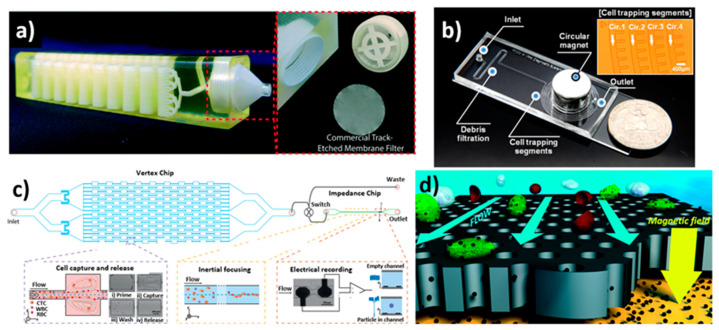
Microfluidic device designs fabricated using various techniques. (**a**) 3D-printed device showing the microchannels with layers and the filter holder; reprinted with permission from ref. [79], 2019, Royal Society of Chemistry. (**b**) Microfluidic device fabricated by photolithography showing spiral channel and cell trapping segments; reprinted with permission from ref. [89], 2018, Elsevier. (**c**) Representation of label-free enumeration of CTCs using a vortex chip connected to an impedance cytometry chip; reprinted with permission from ref. [95], 2019, John Wiley and Sons. (**d**) Schematic of the detection strategy of the micro-aperture chip system for CTC detection; reprinted with permission from ref. [96], 2015, Royal Society of Chemistry.

Molding techniques involving PDMS and other thermoplastics are the most common approaches to fabricating microfluidic devices [74]. The disadvantages of traditional fabrication approaches are that they require a cleanroom, are expensive, utilize time-consuming wafer processes, and require the labor-intensive manual assembly of multiple layers. These factors have limited their wide application [75,76]. Furthermore, it is difficult to efficiently fabricate true 3D structures with large surface areas to increase CTC capture efficiency [77,78]. In recent years, 3D printing, which can create 3D objects layer by layer, has received a lot of attention as a potential replacement for the PDMS-based conventional molding process. In the additive manufacturing (AM) approach, the device is fabricated using a 3D printer and computer-aided design (CAD) software to design the desired shape in a short amount of time. Chu et al. created monolithic microfluidic devices to separate CTCs from whole blood samples [79] (Figure 2a). The fabricated device has a 100 mm channel length, 20.5 mm breadth and 19.2 mm width. The microfluidic device is comprised of two inlets for a sample, a buffer, and an outlet for collecting the waste. The main advantages of this device are that during the filtration process, potential cell damage due to handling the sample was eliminated, and the desired pore size could be attained with high resolution in commercially available membrane filters. Further, Gong et al. developed controlled-compression integrated microgaskets (CCIMs) and simple integrated microgaskets (SIMs), which are bound with small chips to form a wider connection of chips accomplished by a microelectromechanical system (MEMS) and nanoelectromechanical system (NEMS) [80]. SIMs or CCIMs are 3D printed as part of the device’s fabrication. Thus, no additional materials or components are needed to connect to the larger 3D-printed interface chip. Later, Chen et al. developed a microfluidic device with 3D-printed internal structures to facilitate high fluid flow and surface area [81]. The printed structure was functionalised with EpCAM antibodies to capture CTCs.

### 2.2. Etching Technique

Etching is the process of protecting the desired area of a substrate while treating the other in order to remove a particular depth of material. The parts that we do not want to etch are usually protected. Liu et al. used wet etching and thermal bonding to create a pyramid-shaped microfluidic device with one inlet and six outlets [82]. The microchamber is a critical functional component of microfluidic devices for CTC separation. A layer of chemical-corrosion-resistant adhesive tape was pasted on a standard glass slide, and a laser ablation system was used to transfer the desired prototype onto the adhesive tape. The first round of tape was then peeled off, and the glass slide with patterned tape was immersed in the etching solution for 25 min at an etch rate of 1 μm/min. The second and third annular tapes were peeled off, and the glass slide with patterned tapes was dipped in the etching solution for 7 and 8 min, respectively. After the device was completed, a laser was used to punch one inlet and six outlets to allow the blood samples to flow. Each outlet was located on a different layer at different heights of the microfluidic device. The first, second, and third steps were respectively 40, 15, and 4~8 μm high. The device showed a throughput of ~99%. The device has the advantages of being simple to set up, having high isolation efficiency, demonstrating improved throughput and not requiring an expensive capture reagent. Further, Yang et al. reported a wet etching and thermal bonding process to create a unique, low-cost, wedge-shaped microfluidic device made of two glass pieces with appropriate specificity and sensitivity [83]. The device is comprised of two inlets, a linear reservoir, and an outlet. After coating a standard glass slide with a chemical-corrosion-resistant adhesive tape, the laser ablation system was used to transfer the microchannel design onto the adhesive tape. The glass slide coated with patterned tape was immersed in a glass etching solution to create a microchannel with a continuously decreasing height (from 60 to 5 μm). Then, two inlets and an outlet (0.5 mm in diameter) were drilled on the glass slide to obtain the final chip. After a dynamic heating and annealing process in a programmable muffle furnace, the two glass slides were bonded together.

### 2.3. Mold Punching Technique

The fabrication of microstructures via conventional techniques can be costly due to the need for expensive equipment set up and maintenance and the time-consuming nature of the process. If micro- or nano-scale processes can be replicated, manufacturing costs can be drastically lowered. In this technique, micro/nanostructure molds are fabricated once, and products can be duplicated from them. The inverted or negative aspects of the device construction are present in the masters [84]. Liao et al. created an optically induced dielectrophoresis (ODEP) microfluidic device with a T-shaped microchannel made up of four layers: layer A (PDMS), layer B (indium-tin-oxide glass substrate), layer C (double-sided adhesive), and layer D (indium-tin-oxide glass substrate coated with photoconductive material) for the isolation of CTCs using EpCAM/CD45 markers [85]. To facilitate cell suspension transfer, the main channel and side channel’s dimensions (L × W × H) were set to 2500 × 1000 × 60 µm and 2500 × 400 × 60 µm, respectively. The junction area in the T-shaped microchannel that was specified for CTC separation was 1400 × 1000 × 60 µm. The device consisted of three punch holes for tubing connections, with each hole used for loading the sample, harvesting the fresh, waste cell suspension samples, and collecting the separated cells. The advantages of this device included the fact that the cell manipulation process was simpler and easy to operate.

### 2.4. Photolithography Technique

Photolithography has been widely used in the fabrication of microfluidic devices. It entails exposing a photoresist-coated substrate to light so that the selectively developed regions can be protected from/subjected to subsequent fabrication processes like etching or deposition [86,87]. This process, however, necessitates the use of costly photolithographic facilities with specialized lighting for working with ultraviolet (UV)-sensitive materials [88] and uses light-sensitive photoresist to transfer a geometric design from a photomask to a smooth surface. On a glass slide, Kwak et al. reported a spiral-shaped channel microfluidic device [89]. Each circular channel measures 250 µm in width and has a gap between them, with a channel depth of 130 µm. The distance between the spiral channel and the magnet (i.e., radius) was reduced from 3500 µm to 500 µm. High throughput and selectivity are two advantages of this design (Figure 2b). Further, Fan et al., on the other hand, devised a novel size-based separation approach for the rapid identification and isolation of CTCs [90]. The authors created a microfluidic device based on a polydimethylsiloxane (PDMS) membrane filter. The device had a thickness of 60 µm, a diameter of 6.9–10.8 µm, and a gap of 25 µm between two holes. The microfilter produced using lithography has several advantages, including precise, uniformly dispersed pores, high porosity, low cost, and quick processing. However, this method is not suitable for mass production. Later, Yan et al. fabricated an electrochemical microchip for high-efficiency CTC isolation to address the limitations of prior efforts [91]. The PDMS micropillar-array-based electrochemical microchip had hierarchical structures spanning from µm to nm, which were created using a traditional soft lithography approach and then gold layer plating for the electrochemical capture and lysing of captured cells. Similarly, Zhou et al. created a PDMS-based multi-flow microfluidic system using dry film resist instead of SU-8, followed by soft photolithography [62]. The developed straight channel had a length, width, and height of 20 mm, 150 μm, and 50 μm on PDMS, which was bound to a glass slide followed by plasma treatment. 

Kulasinghe et al. designed a multi-flow straight microchannel of 50 µm height and 150 µm width, with two inputs and two outputs for inertial cell migration [92]. The device uses size-dependent separation from the inertial movement of a mixture of WBCs and CTCs, allowing for the isolation of larger CTC clusters as the channel length increases. The sample was injected through the outer inlet, while the phosphate-buffered saline was injected through the inner inlet (PBS). Cells migrated transversely from the sample zone into the clean buffer flow channel as a result of inertial force. Yoon et al. designed a 4.5 × 4.5 cm^2^ microfluidic device on a surface-oxidized silicon chip [93]. The device has two inlets for the sample and the buffer, which are followed by two outlets for waste and isolated cells. The main channel measured 500 μm in length. The slanted weir runs from the upper side of the main channel wall to the branch point. The height of the slanted weir was 7 μm lower than the height of the main channel. A double-layer photolithographic technique was used to pattern the slanted weir-integrated microfluidic channel. Initially, the first layer was spin-coated with a thickness of 23 μm using negative photoresist SU-8 2050, and the second layer with a weir gap was spin-coated with a thickness of 7 μm using negative photoresist SU-8 2007 to get the expected slanted weir-designed device. In other work, Chen et al. fabricated a PDMS-based microfluidic design consisting of gallium nitride (GaN) and aluminium gallium nitride (AlGaN) layers integrated with a field-effect-transistor (FET) chip of 1.2 × 0.8 mm by the plasma etching and metal deposition method followed by a molecular beam epitaxy process [94]. Photoresist SU-8 was spin-coated on a silicon wafer with a thickness of 30 µm; the length, width and height of the channel were set to 60, 20, and 30 µm, respectively. The upper layer of the device was composed of two inlets for cells and buffered saline with four trapping microchambers for cell capturing. The bottom layer was embedded with an FET sensor array on the epoxy substrate. Raillon et al. printed a circuit board coated with a positive photoresist to fabricate a label-free PDMS microfluidic device for the isolation and enumeration of CTCs from human blood samples [95]. At first, a glass wafer was coated with lift-off resist and positive photoresist, followed by printing electrodes using a laser writer to achieve a glass chip with electrodes. Secondly, an SU-8 mold was used to develop a PDMS chip using standard photolithography. The glass chip and PDMS chip were combined to form a single PDMS impedance chip. The channel dimensions were 70, 16, and 40 µm in depth, length, and width, respectively. The device consisted of a plastic vortex chip and PDMS impedance chip, which were connected for fluidic flow. Syringe pumps were used for the injection of the sample into the channel. The flow rate was optimized for the vortex chip at 7 mL/min and reduced to 100 µL/min for the impedance chip. Further, captured CTCs were flushed out with an increased buffer flow rate of 8 mL/min. An excitation voltage was applied between two electrodes at 500 mV and 460 kHz frequency with a flow rate of 100 μL/min, 10 kHz bandwidth, and 100 kHz sampling frequency to detect cancer cells. The advantages of the specific electrode design chip included its high-frequency measurements, ease of fabrication, and fast particle counting (Figure 2c).

Similarly, Chen et al. fabricated a PDMS-based hybrid magnet-deformability CTC chip patterned through a photolithographic technique [97]. The thickness of the silicon wafer post-spin coat was 7 µm, where the CTCs were isolated using a magnetic force. The 12 rows of micro-elliptical pillars were designed within the channel. The distance between adjacent micropillars was gradually reduced from 18 to 5 µm for effective CTC removal, while the width between adjacent arrays remained constant at 1500 µm. The presence of a magnet beneath the device aided in increased the capturing efficiency. The micro-ellipse was comprised of three parts, which include a half-ellipse with a semi-long axis of 30 µm, a half-circle with a radius of 15 µm, a rectangle with a length of 30 µm and a device with a depth of 55 µm. Furthermore, Varillas et al. developed a PDMS-based geometrically enhanced mixing (GEM) microfluidic chip with two layers of SU-8 coating (main channel layer and herringbone mixer layer) for the isolation of CTCs using EpCAM antibodies [98]. For the main channel, the thickness of the SU-8 2035 photoresist was 50 μm. The herringbone mixer layer was formed by adding a second layer of SU-8 after UV light exposure and post-soft baking. A precise arrangement between the main channel and the mixer was maintained to create the herringbone mixer pattern. The inlet and outlet wells were created by punching the holes in PDMS after a second exposure was performed. Shamloo et al. fabricated a new integrated Y-shaped microfluidic device consisting of two subunits, a functional unit and a mixing unit, through SU-8 photoresist patterning and a wet etching process for the immunomagnetic separation of CTCs [99]. The blood samples spiked with CTCs were passed through a 500 µm wide inlet channel. The functional unit and mixing unit had dimensions (L × W) of 12 × 4 mm and 9 × 1 mm, respectively, in which the channel was subjected to an alternative voltage by 10 electrodes arranged in a zigzag pattern. The sample flowed for 3 mm before reaching the diverging region, where it extended for 7 mm towards the outlets. Non-tagged cells were collected through the upper outlet, while magnetic-particle-tagged cells were isolated through the lower outlets, which had a magnet beneath them. The important features of the device were its simple geometry, high efficiency, and high feasibility. However, it was lacking in high performance. 

**Figure 3:**
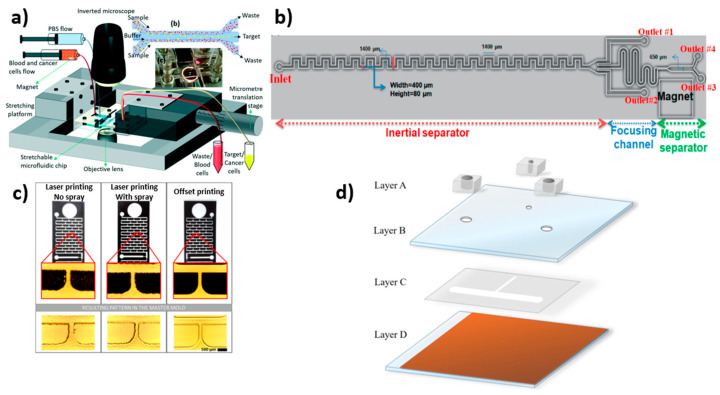
Microfluidic device designs fabricated using various techniques. (**a**) Schematic of the working setup of the stretchable microfluidic device, with an inset showing the multi-flow microchannel with a real stretchable microfluidic device; reprinted with permission from ref. [102], 2021, Royal Society of Chemistry. (**b**) Schematic of hybrid microfluidic cell separation device showing CTC sorting by an inertial focusing microchannel followed by magnetic separation [107]. (**c**) Photomasks fabricated using offset printing showed better resolution and smooth surface over other laser printing techniques [108]. (**d**) Schematic illustration of the optically induced dielectrophoresis (ODEP) microfluidic system assembly where Layer A was composed of fabricated polydimethylsiloxane (PDMS) components; Layer B was composed of indium-tin-oxide (ITO) glass; Layer C was composed of double-sided adhesive tape with microfabricated microchannels; and Layer D was composed of ITO glass substrate coated with a layer of photoconductive material [85].

Chang et al. used a silicon fabrication process to create a PDMS-based microfluidic chip to capture CTCs [96] (Figure 2d). The device was made up of 8 microchips with dimensions (L × W) of 40 × 20 mm. Each chip had a 9 mm by 3 mm porous area in the center, with a pore area thickness of 50 μm. These microchips were covered with a 1 mm thick glass slide. A PDMS layer of ~2 mm thickness was used as a spacer between the glass slide and microchips to form the fluidic chamber. The dimension of the fluidic chamber was defined by a laser cutter with a 30 mm by 3.8 mm grove. The entire setup was placed on an acrylic stand where a magnet was placed. The inlet and outlet were connected to the sample source and peristaltic pump, respectively. This parallel flow micro-aperture chip system has several advantages, including compatibility, ease of use, and the ability to reuse the chip for cell analysis. Later, Chen et al. used soft lithography to create a microfluidic device with a microwell-structured array for the analysis and isolation of targeted tumor cells [100]. The length and depth of the channel were 10 mm and 60 μm, respectively and the width of the chamber was 2.3 mm. The depth and diameter of four various-sized microwell structures were 5.0 µm/18 µm, 5.0 µm/20 µm, 5.0 µm/22 µm, and 8.0 µm/20 µm, respectively, with excellent selectivity for CTCs. Hoshino et al. designed a PDMS-based immunomagnetic microchip for the capture of CTCs from spiked cultured cancer cell lines by magnetic nanoparticles (MNPs) functionalized with EpCAM antibody [101]. UV-patterned SU8-photoresist coated on the silicon wafer was used as a master. The developed microchannel on PDMS was bonded on a glass substrate with a thickness of 150 µm. The developed microchannel measured 30 mm in length, 20 mm in width, and 500 µm in height. Fallahi et al. used photolithography to create a stretchable, flexible microfluidic device for the size-based separation of CTCs [102]. The channel dimensions of the developed device were 100 mm, 100 µm, and 45 µm in length, width, and height, respectively. There were sample and buffer flow inlets as well as waste, large-cell outlets, and small-cell outlets. The entire chip was placed on a specially designed stretching platform (Figure 3a). However, when compared to other size-based microfluidic separation techniques, the device setup was complicated. Further, Jiang et al. demonstrated the use of microbubbles to extract CTCs in a label-free, high-throughput acoustic microstreaming technique [103]. SU-8 2075 photoresist and soft lithography were used to construct the device on a 4-inch silicon wafer. The system was made up of 101 pairs of lateral cavity acoustic transducers (LCATs), each with one inlet and two outlets. The device had a width of 750 µm and was mounted on a piezoelectric transducer with ultrasonic gel between them. The isolation of CTCs by LCATs depended on the oscillation of trapped microbubbles in lateral slanted dead-end side channels to generate a first-order oscillatory flow at the air–liquid interface followed by a second-order streaming flow that consisted of an open microstreaming flow and a closed-looped microstreaming vortex. The dead-end of the channel was tilted at 15° to allow bulk flow through the microstreaming. The narrow gap in the flow area between the looped microstreaming vortex and the air–liquid interface was controlled by the voltage, which regulated the particle size that flowed through. Cells that were smaller than the gap moved forward along the flow by trapping large CTCs. This method allows rapid isolation with the potential to isolate multiple types of CTCs.

Furthermore, Jou et al. used photolithography to create a silicon-based V-BioChip with nano-pillar arrays with a chip dimension (L × W × H) of 32 × 34 × 0.7 mm [104]. A metal-assisted chemical etching technique was used to create nano-pillars within the microchamber. The chip surface was coated with a layer of polyethylene glycol-biotin (PEG-biotin) using a vapor deposition method. Streptavidin was attached to the biotin end using a liquid deposition method to improve capture efficiency. The surface-modified chip with nano-pillars promotes antigen-antibody interaction between the surface and CTCs, resulting in cell capture. Furthermore, Zhang et al. created a label-free microfluidic device for isolating CTCs from breast cancer patients’ blood samples [105]. The photolithography technique was used to develop microchannels to fabricate the microfluidic device. An inlet, a cell intercept area and an outlet were present on the chip. The impurities were filtered through two layers of hexagonal columns in the microchannels. The first and second narrow channels were 50 µm and 20 µm long, respectively, with cell filtration occurring in 30 main channels and 31 side channels. The channels consist of 40 µm cylindrical wells separated by a 100 µm separation distance. Reinholt et al. created a PDMS microfluidic device using photolithography to isolate CTCs using aptamer and extract and to amplify DNA for gene mutation analysis [106]. The device consists of two orthogonal microchannels with two micropillar arrays for CTC isolation at the intersection of the two microchannels and the genomic DNA isolation array downstream of the cell capture array. The cell channel was 1 mm wide, whereas the DNA channel was 500 µm to 1 mm wide and 25 µm deep. Micropillars with a diameter of 50 µm made up the cell capture array. The DNA micropillar array was spaced in a gradient starting at 10 µm and ending at 7 µm. Nasiri et al. developed a hybrid PDMS microfluidic device for CTC isolation via inertial and magnetic separation [107]. For the isolation of CTCs from blood samples, the device consists of an asymmetric serpentine inertial channel, an inertial focusing channel and magnetic cell separation zones. The dimension of the inertial channel was set to 400 µm in width and 80 µm in height, followed by a magnetic separator channel width of 650 µm (Figure 3b).

### 2.5. Printing Technique

Despite the fact that 3D printing is a cheap, robust and scalable method for producing master molds [109,110], there are still challenges that have prevented microfluidic developers from adopting 3D printing, including resolution, throughput and resin biocompatibility [111]. Attempts to reduce the cost of the technique have focused on UV lighting, laser/offset printing, etc. [112,113,114] Laser printing and offset printing could be cost-effective alternatives to expensive photolithography technology. Nguyen et al. investigated methacrylate (MA) gel, a type of nail polish that has been shown to work as a photoresist material instead of SU-8, to develop a master mold with additional benefits such as low cost, rapid production, high resolution (100 µm thickness, 100 µm feature size), high accuracy, and reproducibility [108] (Figure 3c). They used laser and offset printing techniques for photomask generation. The fabricated microfluidic device had a diameter of 100 µm and a height of up to 1 mm. They devised a cost-effective method for fabricating microfluidic devices. To save money, standard procedures like spin coating, plasma etching, and aligners were kept out of the device fabrication. Xu et al. created a microfluidic device out of polymethyl methacrylate (PMMA) by using a laser engraving machine to create microchannels on the surface for CTC isolation [115]. The device was divided into two major components: a filtration system on top and a magnetic microfluidic chip at the bottom. The filtration system used a micropore array membrane to isolate CTCs before filtering out the waste cells. The filter membrane measured 20 × 20 mm and had a pore size of 10 µm. The CTCs with trace WBCs were rinsed off the membrane after filtration for further purification. The magnetic microfluidic chip with a magnetic base of 70 mm with a diameter of 50 mm was used for the negative sorting of CTCs. This device showed low capture efficiency and needed two steps for the isolation of CTCs. Recently, Gurudatt et al. fabricated an electrochemical microfluidic channel modified with conducting polymers by a screen printing approach using carbon ink on a glass slide [116]. The developed microchannel exhibited a width and height of 95 ± 2.5 µm and 15 µm, respectively. The screen-printed channel was dried at 60 °C for two days. Further, the channels were covered using a glass slide. Later, for the amplification of separation, the channel wall was modified with a DAT monomer to covalently attach lipids. Further, Nieto et al. fabricated microchips with pillars on a soda-lime glass substrate using a laser-direct writing technique followed by thermal treatment [25]. An aluminum film was placed on the rear side of the soda-lime glass to increase the ablation. A cylindrical array of micro-posts with 420 µm diameter with a pitch and depth of 245 µm was formed. Further, the pillars were functionalised with EpCAM to facilitate CTC isolation.

### 2.6. Overall Summary of the Fabrication Process

Several fabrication methods have been discussed, each with its own set of characteristics. One must know the minimum feature sizes that the above approaches can produce, as well as a variety of other criteria such as surface roughness, aspect ratio and normal working size, in order to get benefit from the available techniques. Factors such as fluidic outcomes, pressure drop, microchannel, and process time play a major role in the development of the device. Though there are several techniques available for the fabrication of microfluidic devices, photolithography-based devices are determined to be promising in terms of channel dimension precision based on the aforesaid results. The technology, however, can only be used for two-dimensional devices. Additive manufacturing, on the other hand, is cutting-edge, with the potential to create three-dimensional monolithic devices. The main disadvantage is that they are not precise enough for micrometer channels. Therefore, in the near future, 3D printing technology could overcome the challenges and replace the traditional photolithography process for fabricating microfluidic devices. In general, microfluidic devices are fabricated from a variety of materials, including silicon, glass, metals, ceramics, and hard plastics, and they require several fabrication processes, including thermal bonding, chemical etching, and reactive ion etching, which require more time and effort. PDMS-based microfluidic devices, on the other hand, have advantages due to their low cost, optical transparency and biocompatibility.

## 3. Isolation of Circulating Tumor Cells (CTCs) by Microfluidic Devices

During metastasis, cancer cells detach from the primary tumor and intrude apart into tissues in the bloodstream. To detect and isolate CTCs, various techniques including centrifugation, magnetic separation, microchips, filtration, micro/nano substrates and biomarkers have been used. With the widespread adoption of microfluidic techniques, a large number of researchers have worked hard to develop more efficient and reliable CTC separation technologies ranging from immunomagnetic beads to size-based microfluidic devices. Currently, the major commercialised products for CTC separation techniques include the CellSearch system, which uses immunomagnetic beads, and the CelarCellFX1 system, which uses size-dependent isolation [117]. The methods for isolating CTCs are mostly based on biological qualities of the tumor cells, such as specific antigen expression and receptor, or physical properties of the tumor cells, such as size and deformability. Inertial focusing, acoustics, microfluidic filters, optics and dielectrophoresis are some of the size and deformability-based approaches.

### 3.1. Size-Based Isolation

Due to its visibility and ease of management, CTC separation based on size and flexibility is one of the oldest approaches. The principle of separating cells from the main flow channel through filtration is, in fact, rather simple. Membrane devices are designed to act as a filter, allowing blood to flow while separating CTCs based on their size and deformability. When diluted blood travels through the main channel, cells greater than a certain size are captured by this membrane filtration set-up within the device, while smaller cells continue on their course and are separated. The risk of clogging, the requirement for frequent maintenance, cleaning, and the incapacity of cells to recover after filtration are all prevalent issues with these devices. This method’s most serious flaw is that it can’t separate more than one particle type in a single stage. Microfluidic systems capture tumor cells more efficiently via filtering because pore sizes and geometries are carefully controlled by microfabrication. Filters are divided into four types based on their structures: weir-type, pillars, crossflow, and membranes [118]. Size-based filtration using polymer membranes or microsieve membrane filter devices has been shown to extract CTCs from whole blood samples based on the morphological size differences between cancer cells (~15–40 μm in diameter) and leukocytes (~10 μm in diameter) [119]. The size, geometry, and density of the pores in the microfilters can be controlled uniformly and precisely. In addition, this technology can provide maximum sample processing capability via parallel arrays of multiple flow cells, which reduces processing time, cost, and filter clogging while facilitating mass production and high-throughput screening for large-scale clinical studies. 

**Figure 4:**
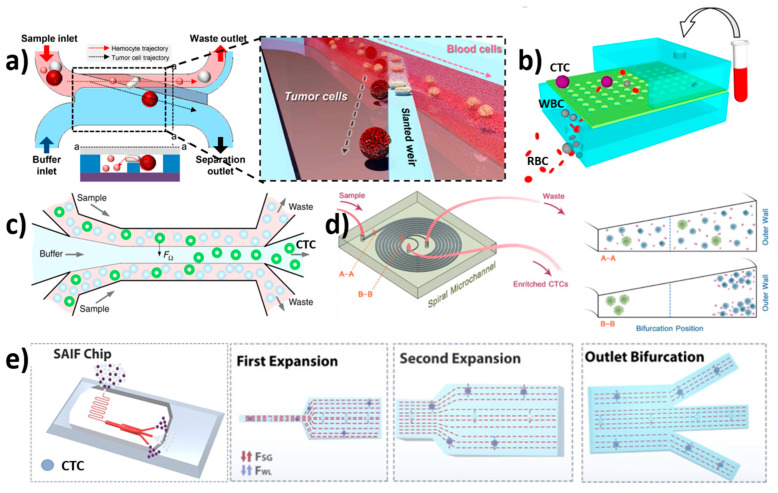
Schematic illustration of microfluidic devices for the isolation of CTCs using various techniques. (**a**) An overview of a slanted weir device; separation of CTCs over a slanted weir based on distinct size and deformability [93]. (**b**) Schematic of the microfluidic device integrated with a PDMS microfiltration membrane for CTC capture; reprinted with permission from ref. [90], 2015, Elsevier. (**c**) Top view of the multi-flow effect of a size-dependent inertial migration microfluidic system representing rotation-induced lift force (FΩ) for the isolation of CTCs [62]. (**d**) Enrichment of CTCs using spiral microfluidic technology utilizing inertial lift force [120]. (**e**) Illustration chip, self-amplified inertial-focused cell bifurcation of CTCs in the microfluidic channel; reprinted with permission from ref. [121], 2020, American Chemical Society.

Yoon et al. developed and reported a slanted weir microfluidic channel to reduce haemocyte contamination during CTC isolation [93] (Figure 4a). With a flow rate of 2.5 mL/h and 3.8 mL/h for a breast cancer cell line (LM2 MDA-MB-231) at 0.8° weir to 0.5° weir, a high separation efficiency of ~97% was achieved. The viability of the collected tumor cells was also determined using the trypan blue assay, and it was found to be 97.1% for the 0.8° weir and 95.8% for the 0.5° weir. The viability of the 0.5° weir was slightly lower depending on the high flow rate and shear rate. This chip showed high separation efficiency with minimal contamination. However, the major drawback was its low throughput. Furthermore, Liu et al. developed a simple pyramid-shaped microchamber that is feasible, cost-effective, and highly efficient for CTC separation from breast carcinoma patients [82]. With an optimised flow rate of 200 μL/min, the capture efficiency of the device was assessed with a fresh blood sample in five sequence concentrations of 25–200 cells/mL using four different cancer cell lines (BGC823, H1975, PC-3, and SKBR3) spiked into DMEM medium. As a proof of concept, polystyrene beads with diameters of 10 μm (red beads) and 20 μm (blue beads) were allowed to pass through the pyramid-shaped channel at a flow rate of 200 μL/min. When the flow rate was increased to 300 μL/min, the capture efficiency increased to 92% and 89%, respectively, at different outlet heights of 6 μm and 8 μm. This method has advantages, including lower sample consumption, a simple experimental procedure, high capture efficiency, and ease of observation. Finally, from the DMEM medium, the SKBR3 cell line had a capture efficiency of 93%, while the healthy blood sample had a capture efficiency of 89%. Further, Fan et al. designed and developed a PDMS membrane filter-based technique for the isolation of CTCs [90] (Figure 4b). At a flow rate of 10 mL/h, >90% cancer cell recovery was achieved from a blood sample spiked with lung cancer cells. Later, Zhang et al. created a label-free microfluidic device for isolating CTCs from breast cancer patients [105]. At a flow rate of 10 mL/h, the device demonstrated 73.6% capture efficiency and an 82% recovery rate. The main and side microchannels were 80 µm and 50 µm and 50 μm and 50 μm in width and height, respectively; the filter microchannel was 40 μm in width 10 μm in height. The device was used to isolate CTC cell strains such as SKBR3, MCF-7, and MDAMB231. Immunofluorescence staining was used to identify the cultured cells.

### 3.2. Inertial Focusing Microchannel-Based Isolation

Inertial focusing is a phenomenon that occurs when suspended particles in a fluid stream migrate across flow lines and arrange themselves in equilibrium positions at specific cross-sectional positions. This behavior is caused by inertial forces within the channel and is controlled by channel geometry and flow conditions [122,123]. This phenomenon occurs in straight channels due to a balance of two dominating forces such as shear gradient inertial lift force (FSL), caused by the curvature of the fluid velocity profile and wall induced inertial lift force (FWL), caused by the particle’s interaction with the nearby wall. The particles are pushed toward the channel walls by FSL, while they are moved away from the walls and toward the channel center by FWL [124,125]. As a result, the particles tend to attain a state of equilibrium where these forces are equal. 

Zhou et al. designed a new multi-flow effect of a size-dependent inertial migration microfluidic (MFM) system for the precise detection and isolation of CTCs from spiked blood samples (H460 and HCC827) [62] (Figure 4c). The separation efficiency and purity of CTCs were obtained to be >99% and >87%, respectively, from CTC-spiked blood samples. At a concentration of 10 cells per 5 mL, the device had an efficiency of >83%. The study showed that the average size of WBCs measured around 9 µm, and the average size of the detected CTCs was 30 µm. Additionally, the channel was examined for isolating CTCs from patient blood samples (stage IV lung cancer). The device has the advantage of having a high recovery rate even at very low concentrations, throughput and sensitivity; it had a disadvantage in terms of its performance and recovery rate due to the significant size overlap between target and non-target cells. Later, Gao et al. designed a label-free CTC isolation microfluidic device utilising the advantage of hydrodynamic forces [126]. The chip has a fishbone-shaped channel, rectangular reservoir and inertial focusing microchannel for CTC isolation. RBCs spiked with U87 cells were injected at a flow rate of 9 µL/min, showing 90% separation efficiency with 84.96% purity. Kulasinghe et al. designed a spiral microfluidic chip for the isolation of head and neck cancer cells (HNCs) [120] (Figure 4d). The chip was tested with patients’ blood samples at a flow rate of 1.7 mL/min. The chip utilises inherent Dean vortex flow along with inertial lift force, which drives smaller hematologic cells towards the outer wall by facilitating the efficient separation of CTCs. The chip showed 54% detection efficiency. Furthermore, Warkiani et al. reported the label-free spiral microfluidic chip for the size-based separation of CTCs from the sample using hydrodynamic forces [127]. At a flow rate of 100 µL/min, the chip achieved ≥85% isolation efficiency. The chip could isolate CTCs from a 7.5 mL sample in less than 40 min. However, stacking three chips together yielded better results by isolating CTCs from a 7.5 mL samples in less than 10 min. Thus, the chip showed high throughput. Later, Ozbry et al. developed a microfluidic chip with a symmetrically curved channel for continuous and high-throughput isolation of cancer cells [128]. The cancer cell lines MDA-MB-231, Jurkat, K562, and HeLa were injected into the curvilinear channel at a curvature angle of 280°. The flow rate was increased constantly from 400 µL/min to 2700 µL/min at an interval of 90 s for each 100 µL increase in the injection volume. The study revealed cell size based on flow velocity. The chip exhibits high viability of >94%. 

Nam et al. fabricated a capillary inserted microfluidic device for the isolation tumor cells via viscoelastic flow [129]. The capillary tube facilitates 3D particle pre-alignment prior to separation. The presence of two outlets facilities the isolation of migrated particles with 5 and 10 µm diameter exhibiting ~99% isolation efficiency. At a flow rate of 200 µL/min, 94% of MCF-7 cells were isolated from leukocytes with 97% purity. Further, Abdulla et al. developed a self-amplified inertial focused (SAIF) microfluidic device for the size-based, high throughput isolation of CTCs [121] (Figure 4e). The device demonstrated a narrow zigzag microchannel connected to expansion sites to enable size-based separation. The tested cancer cells such as lung cancer cells (A549), breast cancer cells (MCF-7), and cervical cancer cells (HeLa) isolation efficiency of ~80%. Che et al. developed label-free, size-based isolation of CTCs using vertex microfluidic chip [130]. At a flow rate of 8 mL/min (for diluted blood) and 800 µL/min (for whole blood); 83% capture efficiency was recorded. Thanormsridetchai et al. developed a spiral microfluidic device for capturing of CTCs [131]. The device with five spiral microchannels (500 µm height, 130 µm width, 5.5 mm length) was injected with samples at a flow rate of 1.0 mL/min. The device showed 90% capture efficiency.

### 3.3. Dielectrophoresis-Based Isolation

Dielectrophoresis with external electric field sources is a quick, simple and well-known technique for manipulating a variety of biological particles within a microchannel [132]. It is also used to separate the movement of distinct cancer cells [133,134]. Cancerous cells could be separated from normal blood cells or the cell sample solution using the dielectrophoresis method based on cell properties such as size, morphology, deformability, mechanical, electrical and magnetic properties [122]. 

Chiu et al. investigated the size-dependent separation of cancer cell clusters using an optically induced dielectrophoresis (ODEP)-based microfluidic system [135]. The device was tested with a human prostate cancer cell line (PC-3) and leukocytes to evaluate its performance. The device could isolate as low as 15 cells/mL with a recovery rate of 41.5%. Overall, the proposed method could isolate CTCs with purity as high as 100% at a sample flow rate of 2.5 μL/min. Thus, the method was found to be promising in the isolation of CTCs with high sensitivity without interference from leukocytes. In another study, Li et al. demonstrated the dielectrophoresis technique using an array of wireless bipolar electrodes for the high-throughput isolations of CTCs [136]. The 32 parallel microchannels with 2950 µm, 200 µm, and 25 µm length, width, and height, respectively, were fabricated using the photolithography technique. The device could throughput 100 µL/h samples with a 39.6 mm^2^ device footprint. Further, Kim et al. developed a dielectrophoresis cell-trapping method for the trapping of cancer cells using a microfluidic device [137]. At a flow rate of 100 µL/min, 92 ± 9% of cells were isolated at the designated location. The technique enables the isolation of very low concentrations of cancer cells from large volumes of samples with high recovery. Liao et al. developed an optically induced dielectrophoresis (ODEP)-based microfluidic device for the isolation of high-purity CD45neg/EpCAMneg cells from the blood samples of cancer patients [85]. To recognize the EpCAM, surface marker-positive CTCs and CD45 surface marker-positive leucocytes were stained using fluorescent dyes. The diameters of PC-3 and SW620 cancerous cells were found to be 20.1 ± 1.5 and 1 µm, respectively. The device demonstrated 100% CTC capture purity in capturing live CD45neg/EpCAMneg cells. The device takes around 4 h for the analysis of 4 mL of sample suspension. The recovery rate of the microfluidic device was found to be 81.0 ± 0.7%.

### 3.4. Magnetic Field-Based Isolation

Magnetic field-derived microfluidic chips are broadly classified as labelled methods and label-free methods of isolation. Positive and negative selection are the two most common methods of labelled magnetic isolation. CTCs can be actively isolated using functionalized magnetic nanoparticles (MNPs) when a magnetic field is applied. Specific antigen-coupled MNPs can bind to specific surface proteins expressions on CTCs, resulting in positive CTC selection [138]. Due to the diversity of cancer cells, CTCs shed from primitive tumors are highly heterogeneous, including epithelial cancer cells such as gastric cancer, mesenchymal cancer cells such as osteosarcoma and other cancer cells such as leukemia. This enables a wide range of antigens to be used to label different CTCs with antiepithelial cell adhesion molecule (EpCAM), which is the most commonly used antigen. On the other hand, negative enrichment of CTCs based on WBC depletion was achieved using anti-CD45 surface antigens because the antigens are particularly expressed on the surface of WBCs [139]. Due to inter-patient and intra-patient heterogeneity in tumor biology, particularly in the case of epithelial-mesenchymal transition (EMT), identifying CTC-specific markers becomes difficult. Meanwhile, label-free magnetic isolation isolates CTCs based on their size difference from hematological cells using magnetic fluids such as paramagnetic salt solutions or ferrofluids as media. 

#### 3.4.1. Immunomagnetic (Label)-Based Isolation

Chang et al. developed a novel parallel flow micro-aperture chip system for CTC isolation in the spiked MCF-7 cell line at a flow rate of 2 mL/min [96]. CTCs with sizes ranging from 10 to 30 μm were found in the sample solution after it had been coated with antibody-mediated magnetic beads. The chip detected approximately 89% of the spiked MCF-7 breast cancer cell lines. The device has several advantages, including its ease of use, robustness, compatibility and versatility. The device was integrated with a PDMS microfiltration membrane for CTC capture and a parallel flow micro-aperture chip system for capturing CTCs. Furthermore, clinical samples revealed the possibility of isolating cancer cells (non-small-cell lung cancer cell line and pancreatic cancer cell line) that were bound on beads and captured on the chip’s surface. Furthermore, Kwak et al. investigated the selectivity and capture efficiency of the developed spiral-shaped channel device for two types of tumor cell lines, MDA-MB-231 and MCF-7, based on the level of EpCAM antigen expression [89]. The results showed that the capture efficiency of MDA-MB-231 and MCF-7 cells were 81.2 ± 3.5% and 96.3 ± 1.5%, respectively, at a flow rate of 150 µL/min. MDA-MB-231 cells had an average purity of 82.8%, while MCF-7 cells had an average purity of 85.9%. However, because of the low EpCAM expression in this reported device, several heterogeneous CTCs could not be detected and quantified. Recently, Kang et al. developed a positive and negative method for the isolation of CTCs (MDA-MB-231, PC-3, SKBR3, and MCF-7) by lateral magnetophoresis using magnetic nanobead-functionalized EpCAM and CD45/CD66b antibodies [140]. The lateral magnetophoresis technique was used to design a disposable chip with a microchannel on a multipurpose substrate fixed to ferromagnetic wires. The device works both on positive and negative methods for the isolation of CTCs using anti-EpCAM and anti-CD45/CD66b nanobeads. The ferromagnetic wires were inlaid at 5.7° towards the flow direction on the substrate. As the blood flowed through the lateral magnetophoretic microchannel, the residual magnetic nanobeads were bound to the ferromagnetic wires. The silicon-coated polymer film with a thickness of 12 µm was bonded to a microstructure PDMS replica to form a disposable microchannel substrate. The flow rate and suction rate for the sample and buffer were optimized in the positive method to 2 mL/h and 3.2 mL/h, respectively, resulting in the release of CTCs in the outlet at a flow rate of 0.8 mL/h. This device was evaluated for the isolation of the SKBR3 and MCF-7 cell lines, and the recovery rates were 93.9 ± 1.0% and 98.4 ± 1.5%, respectively. However, this method resulted in low EpCAM expression in MDA-MB-231 and PC-3 cells. Further, the flow rate for the sample and buffer was optimized to 2.8 mL/h for the negative method. The method yielded recovery rates of 85.2 ± 4.2 and 80.7 ± 7.6% for SKBR3 and MCF-7 cell lines, respectively, and 91.0 ± 2.0% and 75.7 ± 9.3% for MDA-MB-231 and PC-3 cells, respectively. A fluorescence microscope was then used to enumerate WBCs and CTCs from the outlet. The positive method produced more pure isolated CTCs than the negative method. Following this, Chen et al. developed a size-based microfluidic device with high capture efficiency for CTC isolation [97]. A few strong permanent magnets were fixed beneath the glass substrate to capture the magnetized CTCs. Three different cancerous cell lines (HCT116, SW480, and MCF-7) were tested with different EpCAM antibody expression levels to evaluate the device. Capture efficiency for MCF-7, HTC116, and SW480 was found to be up to 97.2 ± 6%, 85.7 ± 14.3%, and 91.5 ± 8.9%, respectively. Due to cell line accumulation, capture efficiency was decreased. The flow rate was optimised to 1.5 mL/h for the system operated without a magnet, which showed a capture efficiency of around 90%. The magnetic bead at a high processing rate of 3 mL/h showed a capture efficiency above 90% within 20 min. The live/dead assay revealed 96% cell viability. The reverse flushing process removed the majority of the CTCs from the channel. Despite the device’s high processing rate, there was a lack of capture efficiency. 

**Figure 5:**
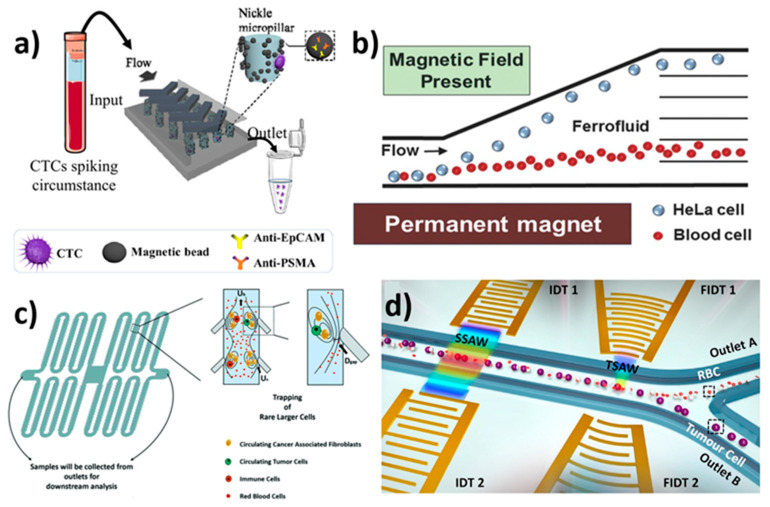
Schematic illustration of microfluidic devices for the isolation of CTCs using various techniques. (**a**) Schematic of the working mechanism of a dual-antibody-functionalised microfluidic device for the isolation of CTCs using magnetic beads; reprinted with permission from ref. [143], 2018, American Chemical Society. (**b**) Schematics of label-free isolation of HeLa cells in ferrofluids under magnetic fields by magnetic buoyancy forces; reprinted with permission from ref. [144], 2015, Wlsevier. (**c**) Schematic of CTC isolation in bubble-based acoustic microstreaming, which releases smaller cells by trapping larger CTCs; reprinted with permission from ref. [103], 2020, Royal Society of Chemistry. (**d**) Schematic illustration of a multi-stage device consisting of a pair of IDTs and FIDTs to generate SSAWs and TSAWs for the isolation of CTCs; reprinted with permission from ref. [151], 2018, Elsevier.

Furthermore, Shamloo et al. created a PDMS-based integrated microfluidic platform for CTC capture using an immunomagnetic technique [99]. The separation and mixing units, as mentioned in the fabrication section, use electric and magnetic forces for high throughput to increase the purity and capture efficiency in the microfluidic system. To evaluate the device’s capture efficiency, anti-EpCAM functionalized iron nanoparticles were tagged to different types of blood samples spiked with 100,000 cancerous cells, such as SKBR3 (human breast cancer cell line), PC-3 (prostate cancer cell line) and Colo205 (colon cancer cell line). The capture rate for SKBR3 and Colo205 cell lines was up to 97%, while the PC-3 cell line was 107%. As a result, this integrated microfluidic device has high compatibility and feasibility in cancer research. Later, Poudineh et al. developed magnetic raking cytometry to generate a phenotypic expression of captured CTCs [141]. The device consisted of circular nickel micromagnets with an array of X-shaped structures. The size of the micromagnets was increased along the channel to enhance the CTC capture efficiency. CTCs coated with anti-EpCAM-functionalised immunomagnetic beads were retained at the capture zone of the device. In addition, Poudineh et al. reported a microfluidic approach for profiling functional and biochemical phenotypes of CTCs [142]. The device consisted of four capture zones with an X-shaped morphology and a single-cell isolation area. The aptamer-coated CTCs functionalised with MNPs were captured at four capture zones by EpCAM expression. This was followed by releasing them to a single-cell isolation area using antisense DNA. The device showed 79 ± 4% recovery efficiency. Recently, Yin et al. constructed a dual-antibody (PSMA and EpCAM)-functionalised microfluidic device for the isolation of CTCs [143]. The dual-antibody-functionalised strategy showed a significant increase in the capture efficiency for LnCAP and LnCAP-EMP cancer cell lines. The device consists of antibody- and Fe3O4@microbead-functionalised Ni (nickel) micropillars under external magnetic conditions and a chaotic herringbone platform (Figure 5a). The device could successfully identify CTCs from 20 out of 24 blood samples.

#### 3.4.2. Label-Free-Based Magnetic Isolation

Zhao et al. demonstrated size-based ferrohydrodynamic HeLa cell isolation using a microfluidic device [144]. Cell mixtures (HeLa cells, blood cells) and ferrofluids were mixed, then injected at a flow rate of 8 µL/min. The magnetic buoyancy force caused deflections of cells from their laminar flow patterns when the magnet was placed close to the channel. The force operating on cells inside ferrofluids is a body force proportional to cell volume, resulting in the spatial separation of cells of various sizes at the microchannel’s end. As a result, larger HeLa cells and smaller blood cells emerge through distinct pathways (Figure 5b). The device exhibited >99% capture efficiency. The method was found to be cost-effective with high throughput. Furthermore, Zhao et al. used label-free size-based ferrohydrodynamic CTC isolation using a microfluidic device [145]. The device showed a high throughput of 6 mL/h with a recovery rate of 92.9%. The device could isolate CTCs as low as ~100 cells/mL. In addition, the device demonstrated recovery rates for cancer cells line such as H1299 (92.3%), A549 (88.3%), H3122 (93.7%), PC-3 (95.3%), MCF-7 (94.7%), and HCC1806 (12.2%). The device showed short-term cell viability, normal proliferation, and unaffected key biomarker expression. Later, Zhao et al. developed a label-free isolation method using ferrofluids to separate low-concentration cancer cells from cell culture lines in microfluidics [146]. The isolation depended on the variation in size of CTCs with WBCs in biocompatible ferrofluids. At a throughput of 1.2 mL/h, the device showed isolation efficiencies of 80 ± 3%, 81 ± 5%, 82 ± 5%, 82 ± 4%, and 86 ± 6% for A549 lung cancer, H1299 lung cancer, MCF-7 breast cancer, MDA-MB-231 breast cancer, and PC-3 prostate cancer cell lines, respectively.

### 3.5. Acoustic-Based Isolation

An acoustic wave is a form of a mechanical wave that propagates across a longitudinal wave and is generated by mechanical stress from a piezoelectric transducer. Surface acoustic waves (SAWs) and bulk acoustic waves (BAWs) are the two forms of acoustic waves. Both have been widely employed in the field of microfluidics to manipulate micro-objects [147]. Travelling SAWs (TSAWs) and standing SAWs (SSAWs) are the two types of SAW-driven microfluidics. SAWs that propagate in one direction and radiate away from acoustic sources are known as travelling surface acoustic waves (TSAWs). Two opposing travelling SAWs interfering or a reflecting travelling SAW create stationary nodes and antinodes in an open or limited domain, resulting in standing surface acoustic waves (SSAWs). Alternatively, bulk acoustic waves (BAWs) are standing waves that propagate within the microchannel’s resonant chamber. To generate BAWs, a piezoelectric transducer is bonded to the microchannels and actuated by an AC power supply in BAW-based microfluidic devices. Unlike SAWs, which propagate along the material’s surface, bulk acoustic waves propagate within the material’s core. As a result, BAW-based microfluidic devices require more energy to create identical acoustic effects to SAW-based microfluidic devices [148,149].

Jiang et al. used the LCATs technique for the isolations of CTCs from breast cancer patients with different stages of cancer [103] (Figure 5c). The advantage of LCATs was their ability to pump samples and trap CTCs without the use of a syringe pump. The device captured 230,000 cells with 200 pairs of dead-end side channels at 6 VP-P (peak-to-peak voltage) and 5.2 kHz, with an average of 1150 cells per pair of dead-end side channels. In less than 8 min, the device could process 7.5 mL of blood samples. However, the real CTC-spiked blood samples showed a capture efficiency of 92.8% with 90% viability. As a result, the technique must be improved in order to achieve higher capture efficiency in real-time applications. Wu et al. examined the acoustic separation of CTCs from leukocytes [150]. A piezoelectric substrate bound to a pair of interdigital transducers (IDTs) in a microfluidic channel generated two Rayleigh waves in opposite directions, resulting in periodic wave nodes and antinodes. In order to facilitate high throughput, a PDMS-glass hybrid channel was used to produce acoustic waves. At a throughput of 7.5 mL/h, 86% CTCs were recovered from the sample. Furthermore, Wang et al. developed a multi-stage device consisting of a pair of interdigital transducers (IDTs) and focused interdigital transducers (FIDTs) using microelectromechanical systems (MEMS) for the separation of CTCs by SAWs [151]. The acoustic waves generated by IDTs enabled the cells to be placed at pressure nodes (Figure 5d), whereas acoustic waves generated by FIDTs push the RBCs from CTCs, resulting in isolation. At a flow rate of 0.3 µL/min, the device showed ~90% isolation efficiency for U87 glioma cells. Karthick et al. developed the acoustic impedance size-independent isolation of CTCs using a microfluidic device [152]. At an optimized flow rate, the device could recover 86% of HeLa cells and 88% of MDA-MA-231 CTCs. Later, Xue et al. presented an acoustic multifunctional micromanipulation (AMM) microstreaming device capable of patterning, tapping, isolating, and rotating microparticles with respect to size and shape [153]. A microcavity array with an inner micro vortex and outer micro vortex was generated by acoustic waves to achieve cell manipulation. The device showed ~90% isolation efficiency. Recently, Cushing et al. reported continuous-flow acoustophoretic negative selections of WBCs from CTCs with the help of negative acoustic contrast elastomeric particles (EPs) functionalised with CD45 antibodies [154]. EP-bound WBC aligned at the channel wall, enabling unbound CTCs to flow through the channel centre. The device facilitated the isolation of label-free CTCs from WBCs with a recovery rate of ~85–90%.

### 3.6. Combined Method-Based Isolation

The combination of two or more modes of isolation techniques in a microfluidic device facilitates the highly efficient isolation of CTCs. Nasiri et al. fabricated a hybrid microfluidic system that uses inertial flow and magnetophoresis to isolate CTCs [107]. The MCF-7 cells were conjugated with EpCAM antibodies and MNPs to improve magnetic susceptibility. These surface-modified cells were mixed with blood cells and were injected into the hybrid device at a flow rate of 1000 µL/min. The device exhibited a separation efficiency of ~95% with a purity of ~93%. Furthermore, Raillon et al. combined a vortex chip and an impedance chip to create microfluidic devices for label-free, high-throughput CTC isolation and enumeration [95]. Firstly, a vortex chip was used to purify the cancer cells. Later, an impedance chip with a pair of electrodes measured the fluctuation of an applied electric field in the presence of CTCs. This device was subjected to beads and tumor cells as proof of concept. PEEK/Tefzel tubings were used to form connections along with the vortex chip, impedance chip and syringe-containing samples. In the vortex chip, the flow rate to capture CTCs was optimized to 100 µL/min. The channel was validated with 8, 15, and 20 µm fluorescent beads through which the vortex chip enriched beads with an amplitude ranging from 250 nA to 100–250 nA. By using an impedance chip, 1477 beads were detected, and 1294 beads were enumerated from the device. Finally, MCF-7 cells were assessed in the channel at an optimized flow rate of 100 μL/min. RBCs and PBMCs (peripheral blood mononuclear cells) were separated using Ficoll. Thus, it was observed that at 60 nA, 95% of MCF-7 cells were separated from RBCs and PBMCs by leaving 5% of MCF-7 as a false negative. Later, Shamloo et al. employed a passive and a hybrid centrifugal device design to isolate tumor cells with the help of MNPs [155]. In the passive design, a contraction–expansion array (CEA) microchannel with a bifurcation region was used to isolate tumor cells through inertial effects and bifurcation law. In the hybrid design, a CEA microchannel with stacks of magnets was used to isolate magnetically labelled tumor cells. The devices were utilised to isolate human breast cancer cells (MCF-7). The devices were performed with various centrifugal speeds, demonstrating a recovery rate of 76% at 2100 rpm for the passive design. On the other hand, the hybrid design showed an 85% recovery rate at 1200 rpm. Though the hybrid design showed a high recovery percentage, the passive design was less space-, cost-, and time-consuming.

**Figure 6:**
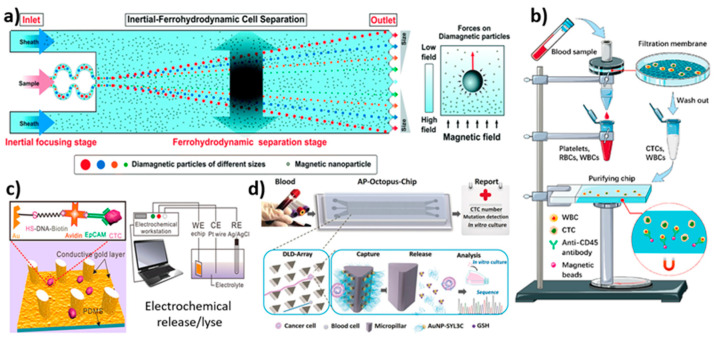
Schematic illustration of microfluidic devices for the isolation of CTCs using various techniques. (**a**) Schematic illustration of working principle of an inertial-ferrohydrodynamic cell separation chip in ferrofluids under a magnetic field; reprinted with permission from ref. [158], 2021, Royal Society of Chemistry. (**b**) Schematic of isolation of CTCs through filtration, followed by anti-CD45 antibody functionalized magnetic beads [115]. (**c**) Schematic illustration of e-chip exhibiting a conductive gold layer functionalised with EpCAM antibodies responsible for the capture and electrochemical release/lyse of CTCs; reprinted with permission from ref. [91], 2017, American Chemical Society. (**d**) Schematic of DLD working principle of AP-Octopus-Chip, where CTCs interact with micropillar-functionalised AuBO-SYL3C to get captured and released by Au-S bond disruption; reprinted with permission from ref. [161], 2019, John Wiley and Sons.

Chen developed a triplet microchannel spiral microfluidic chip that interconnected with many tilted slits based on inertial and deformability principles for the continuous isolation of CTCs [156]. Using inertial and viscous drag forces, cells of various sizes were made to achieve different equilibrium throughout the microchannel. The bigger CTCs were gathered at the central streamline. The chip showed a high isolation capacity of 90% at a flow rate of 80 mL/h. Later, Antfolk et al. fabricated a microfluidic device with two inlets and three outlets for the label-free, on-chip separation and enumeration of target tumor cells [157]. They bound together acoustic and dielectrophoresis chips through plasma treatment. The outlet of the acoustic chip was aligned to an inlet of the dielectrophoresis chip for the efficient isolation of target cells. Prostate tumor cells (DU145) were effectively isolated from peripheral blood mononuclear cells at a recovery rate of 76.2 ± 5.9%. Furthermore, Liu et al. designed a label-free inertial-ferrohydrodynamic CTC-capturing microfluidic device [158] (Figure 6a). The technique enabled the high-throughput, high-resolution isolation of CTCs. The method could differentiate the ~1–2 μm diameter difference in cells for efficient separation. The developed method showed a recovery rate of 94% with high purity. In addition, Xu et al. created an integrated microfluidic device for CTC isolation [115]. The prefiltered CTCs were subjected to magnet-assisted isolation on a microfluidic chip comprised of anti-CD45 antibody-functionalized magnetic beads (Figure 6b). For PC-9-spiked blood samples, the device demonstrated a capture efficiency of ~85% and a purity of 60.4%. Despite the fact that the method involved two steps of isolation with high throughput and minimal cell damage, the device lacked capture efficacy. Later, Garg et al. presented a multi-functional microfluidic microstreaming LCAT-based device for the size-based isolation, enrichment, and in situ biomarker-based sorting of cells from blood [159]. At a flow rate of 25 µL/min, targeted MCF-7 cells were trapped in microstreaming vortices at ~100% efficiency.

### 3.7. Electrochemical-Based Isolation

Electrochemical detection relies on the transfer of electrons at the analyte-electrode interface, which is frequently accompanied by the process of analyte-receptor recognition. Electrochemical procedures have a fast response time, cheap cost, simplicity, clinically appropriate sensitivity, specificity and the potential to miniaturize when compared to other analytical methods [160]. Meanwhile, they are frequently used in conjunction with other technologies to achieve multimode detection with increased accuracy and sensitivity.

Yan et al. fabricated a micropillar array electrochemical microchip for the isolation and analysis of CTCs [91]. The device surface was coated with a gold layer, followed by oligonucleotide modification via gold-thiol (Figure 6c). Further, avidin and EpCAM antibodies were functionalised. In order to lyse the cells, the device was modified with two slices of gold to use as the working electrodes. By applying a voltage, the captured cells were lysed. The –OH ions generated during electrochemical lysis broke down the lipid bilayer of the captured cells. The device showed a capture efficiency of 85–100%. Furthermore, Gurudatt et al. developed an electrochemical microfluidic system that combines CTC separation, enrichment, and detection [116]. Whole blood cells flowing through a microchannel were initially functionalized with electroactive daunomycin (DM, an anticancer drug that can selectively interact with CTCs). The target species in the microfluidic channel exhibited a wave-like motion when an alternating current perpendicular to the hydrodynamic flow was applied and was segregated and enriched in a size-dependent manner. The CTCs were subsequently examined using a direct DM oxidation method with an electrochemical sensor at the channel end. With a separation efficiency of 92.0 ± 0.5% and a detection rate of 90.9%, this device is capable of successfully discriminating various cancer cells in patients’ blood samples.

### 3.8. Biological Interaction-Based Isolation

**Figure 7:**
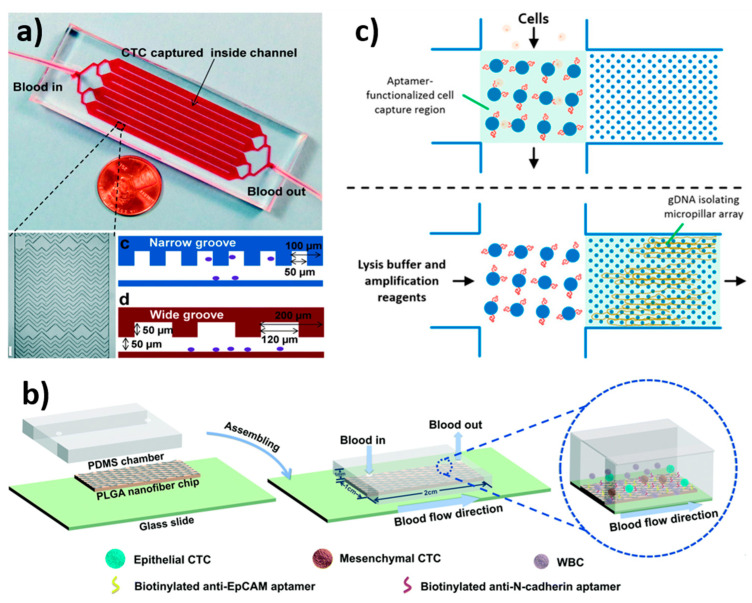
Schematic illustration of microfluidic devices for the isolation of CTCs using various techniques. (**a**) GEM chip with eight parallel channels with an inlet and an outlet showing asymmetric herringbone grooves inside the channel; reprinted with permission from ref. [163], 2013, Royal Society of Chemistry. (**b**) Schematic of dual aptamer-functionalised PLGA nanofiber-based microfluidic chip for the isolation of various phenotypic CTCs; reprinted with permission from ref. [164], 2021, Royal Society of Chemistry. (**c**) Schematic of microchannel design with aptamer-modified micropillar array for capturing cancer cells and isolating their gDNA; reprinted with permission from ref. [106], 2018, American Chemical Society.

Though CTCs are found in the bloodstream, they retain the characteristics of their original tumor cell from the metastatic sites. The expression of EpCAM is a pervasive biological property of CTCs. As a result, EpCAM was used as a specific biomarker for CTC isolation in positive selection. However, the EpCAM protein is present on CTCs but not on blood cells. Thus, other markers such as CD1513, CD6647, and CD45 are used as specific biomarkers for blood cells for negative selection. Stott et al. developed a herringbone microfluidic device by photolithography [162]. The microchannels were functionalised with EpCAM antibodies to facilitate CTC isolation. The presence of a herringbone pattern generates micro vortices, which results in thorough mixing of blood samples, facilitating the high interaction between the functionalised channel surface and CTCs. The device could isolate CTCs from patients’ blood with advanced prostate and lung cancer with a success rate of 93%. The device showed high throughput and promising results. Later, Song et al. developed an aptamer-tailed octopus chip (AP-Octopus-Chip) for capturing CTCs [161]. To improve capture efficiency, a deterministic lateral displacement (DLD)-patterned microfluidic chip was altered with multivalent aptamer-functionalized nanospheres (Figure 6d). CTCs were forced to transverse streamlines and interact with AuNP-SYL3C modified micropillars. Blood cells that are smaller than CTCs stayed inside the initial flow streamline, and bigger CTCs interacted with them. The enriched CTCs were released after capture when the -AuS bond was broken by excess glutathione. Sheng et al. developed a geometrically enhanced mixing (GEM) chip for the capture and isolation of CTCs from pancreatic cancer cell lines [163] (Figure 7a). Initially, anti-EpCAM was biotinylated and loaded to the surface of a microfluidic channel containing L3.6pl, BxPC-3, and MIAPaCa-2 cells in order to capture CTCs. Flow cytometry results show that L3.6pl cells bind strongly to anti-EpCAM, whereas MIAPaCa-2 cells do not. For capturing CTCs, the flow rate and velocity were optimised to 1 μL/s and 0.75 mm/s, respectively. The GEM chip detected ~23 CTCs from 7.5 mL of blood, with the capture efficiency of 90 ± 2% for the L3.6pl cells line and 92 ± 4% for the BxPC-3 cells. The device has the advantage of being able to isolate CTCs with sufficient throughput in 17 min. Overall, the device achieved >90% capture efficiency, >84% purity and a throughput of 3.6 mL of blood in 1 h. However, the device falls short in terms of CTC capture purity. Furthermore, Nieto et al. developed a soda-lime glass-based microfluidic device by using the laser-ablation direct writing method and laser-assisted thermal treatment for the isolation of CTCs [25]. With this treatment, the roughness, optical transparency and reshaping of the microstructures were improved. The surface-modified microchannel with EpCAM antibodies developed by this approach trapped the CTCs. The results showed a capture efficiency of ~76% for HEC-1A tumor cells.

**Figure 8:**
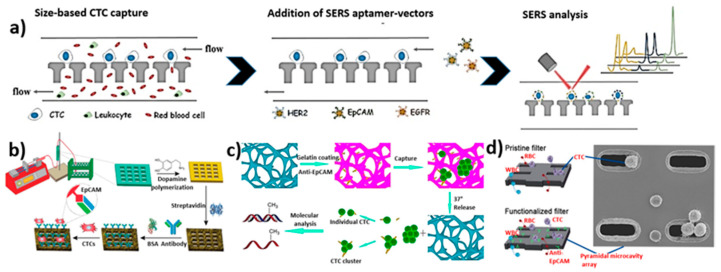
Schematic illustration of microfluidic devices for the isolation of CTCs using various techniques. (**a**) Working strategy of SERS nano vectors for CTC capture, cell phe-notype profiling and multivariate analysis for in situ profiling of CTCs; reprinted with permission from ref. [166], 2018, John Wiley and Sons. (**b**) Schematic of the working setup of the microfluidic platform and surface modification of 3D-printed microfluidic device with an-ti-EpCAM antibody for the isolation of CTCs; reprinted with permission from ref. [81], 2020, Elsevier. (**c**) Schematic surface modification of 3D Ni foam scaffold with gelatin and anti-EpCAM to capture CTCs; these were released at 37 °C for molecular analysis; reprinted with permission from ref. [167], 2017, American Chemical Society. (**d**) Schematic of CTC isolation by a filtration chip functionalised with anti-EpCAM antibody and SEM image of captured cells on pyramidal MCA; reprinted with permission from ref. [170], 2019, Elsevier.

In addition, Jou et al. created the V-BioChip for isolating SKOV3 ovarian tumor cells from epithelial ovarian cancer patients’ blood samples [104]. Using anti-EpCAM antibody interactions on the device’s surface at a flow rate of 0.6 mL/h, the device demonstrated a capture efficiency of 48.3%. The combination of anti-EpCAM antibody and anti-N-cadherin antibody on the device surface resulted in a capture efficacy of 89.6%. Despite the functionalised surface, the obtained results showed a lower capture efficiency. Further, Wu et al. created a PLGA nanofiber, aptamer-functionalized microfluidic device for isolating ovarian cancer cells such as A2780 and OVCAR-3 cells [164] (Figure 7b). The EpCAM-functionalised chip demonstrated a good capture efficiency of 89% for OVCAR-3 cells, while NC3S demonstrated high efficiency of 91% for A2780 with a release efficiency of 88% and 92%, respectively. Later, Reinholt et al. developed a PDMS microfluidic system to isolate HeLa (cervical cancer cell line) and CAOV-3 (ovarian cancer cell line) cancer cells [106] (Figure 7c). For the capture of CTCs via a streptavidin–biotin conjugation, the microchannel surface was functionalized with a DNA aptamer. The capture efficiency was great when the CTCs were suspended in PBS buffer and flushed into the microchannel at a flow rate of 5 µL/min. The collected cells were also lysed using a DNA array channel. The cellular contents were allowed to flow out while the gDNA was isolated on the micropillar. The use of gDNA allows for the extraction of enormous amounts of data from a small number of cells without the need for genome amplification. In another study, Pulikkathodi et al. developed an AlGaN/GaN high-electron-mobility (HEMT) biosensor array for the detection and isolation of CTCs [165]. Furthermore, these chips are mounted on a thermos-curable polymeric substrate. The formed array has several aptamer-immobilized areas, which are sensitive to CTCs. The device showed high sensitivity and selectivity, making it a potential device for CTC isolation. Zhang et al. combined a size-based microfluidic device with surface-enhanced Raman spectroscopy (SERS) for the detection of tumor cells [166] (Figure 8a). Three kinds of SERS aptamer nano vectors were utilised for the detection of breast cancer cell lines in accordance with surface protein expressions. Initially, at a flow rate of 1 µL/min, tumor cells were separated through filtration. Then, SERS receptors were used to analyse the captured CTCs. Recently, Chen et al. developed a 3D-printed microfluidic device for the isolation of CTC from a blood sample [81]. The channel surface was functionalised with EpCAM antibodies to capture EpCAM-positive cancer cell lines, such as MCF-7, SW480, PC-3, and EpCAM-negative 293T cells (Figure 8b). At a flow rate of 1 mL/h with a 2 cm channel length, the device showed a capture efficiency of up to ~92% for MCF-7, ~87.74 for SW480, and ~89.35 for PC-3.

Cheng et al. designed and developed a 3D scaffold microfluidic device with a thermosensitive coating for the isolation and release of CTCs [167]. Gelatin hydrogel was coated on the surface of Ni (nickel) foam. In addition, the surface of the gelatin was functionalised with an anti-EpCAM monoclonal antibody to capture MCF-7 cells (Figure 8c). At an optimised flow rate of 50 µL/min, CTCs were captured. Further, the chip was transferred to an incubator at 37 °C in order to dissolve the gelatin hydrogel to facilitate the release of captured CTCs. The chip showed ~88% capture efficiency. The isolation of platelet-covered CTCs is extremely difficult due to the masking of surface epitopes. Furthermore, Jiang et al. designed a herringbone macromixing microfluidic platform using stealth CTCs as surface markers for the isolation of CTCs [168]. They used epithelial and mesenchymal phenotypes for the platelet-targeted isolation of CTCs. At first, the free platelets were isolated by hydrodynamic size-based isolation. Further, EpCAM/CD41 antibodies were employed for the isolation of platelet-covered CTCs. The device isolated 66% of lung, 60% of breast, and 80% of melanoma cancer cells. Zeinali et al. demonstrated the integrated immunoaffinity-based isolation of CTCs from pancreatic cancer patients [169]. The device could isolate epithelial and epithelial-to-mesenchymal transition CTCs simultaneously by using EpCAM and CD133 antibodies. At a flow rate of 1 mL/h, the device showed ≥97% CTC recovery with >76% purity. Yin et al. designed a micruifluidic device with a silicon filter with a pyramidal microcavity array (MCA) for the isolation of CTCs [170]. In order to improve the capture efficiency, the surface of the MCA filter was modified with an anti-EpCAM antibody (Figure 8d). The device showed a capture efficiency of ~80% for MCF-7, SW620, and HeLa cell lines spiked in whole blood. The device could effectively filter various sizes of CTCs with high capture efficiency. Kermanshah et al. applied magnetic ranking cytometry (MagRC) to a biologically relevant study [171]. Nickel micromagnets of different sizes were developed to create isolation zones to capture magnetized CTCs. The blood samples of mice containing prostate cancer cells were mixed with EpCAM antibody-modified MNPs and were analysed using the MagRC device. Furthermore, Sun et al. developed a size-based separation where the microfluidic device has ~103 pores/mm^2^, exhibiting 68,000 effective pores with a pore diameter of 8 µm [172]. The capture efficiency for MCF-7 cells on the device was found to be 72 ± 10.6% when using the traditional ISET (isolation by size of epithelial tumor cell) technique at a flow rate of 1 mL/min, whereas the capture efficiency of M-ISET (microbeads assisting ISET) was found to be 93.3 ± 3%. As a result, the M-ISET method was found to be a powerful tool for improving the efficiency of CTC separation.

### 3.9. Overview of Microfluidic Device Performance for the Isolation of Circulating Tumor Cells

Importantly, there are two types of CTC isolation methods: physical and biological. Physical approaches are typically based on physical properties, such as size, volume, deformability, density, dielectric properties, and viscosity, with benefits such as high capturing efficiency, simple sample preparation, and cost-effectiveness. On the other hand, biological approaches are based on antigen-antibody interactions. The main disadvantage, in this case, is that it is an expensive and time-consuming method. In addition, there are some challenges and drawbacks in identifying and separating CTCs. When dealing with microfluidic devices, five different technological criteria are to be considered: the detection limit, capture speed, biocompatibility, purity, and high throughput. There are various devices mentioned, such as spiral-shaped, slanted weir, T-shaped microchannel, and multi-flow microfluidic (MFM) systems, geometrically enhanced mixing (GEM) chips, PDMS-based integrated microfluidic platforms, pyramid-shaped microchambers, ODEP-based microfluidic devices, parallel flow micro-aperture chip systems, a label-free microfluidic device for the detection and separation of CTCs with different capture efficiency. Table 1 summarizes the details of microfluidic devices for CTC isolation.

## 4. Conclusions and Prospects 

CTCs play an important role in cancer metastasis and are studied clinically for cancer prognosis and diagnosis, known as liquid biopsy. Though there are several commercial technologies available such as CellSearch, CytoQuest, GILUPI CellCollector, ApoStream, Screencell, and ISET for CTC isolation, these technologies still have some drawbacks to their application in clinics. These devices have tedious fabrication and operational protocols, resulting in limited batch fabrication for large-scale production. In addition, they lack the sensitivity to satisfy clinical demands due to the presence of various kinds of tumor cell types. Hence, there is a need for greater effort to develop CTC isolation techniques. The developed CTC isolation technology should be easy to fabricate and operate. The detection strategy should be simple, fast, and accurate. In this regard, microfluidic technology is a multidisciplinary research field that can be used for capturing and isolating CTCs due to their numerous advantages over traditional separation techniques. When compared to traditional macro-scale devices, the microfluidic technique has numerous benefits, including portability, improved sensitivity, lower operating costs, and higher throughput. 

We summarized perspectives on the various strategical microfluidic devices regarding both label-free isolation and label-based detection of CTCs using various methods such as dielectrophoresis, inertial migration, the electrochemical method, the M-ISET method, the hydrodynamic method, the sandwich moulding method, deformability-based separation, the label-free immune separation method, and the label-based method in this review. Microfluidic devices for physical approaches can be easily fabricated due to their reusability and the absence of expensive antibodies. Thus, physical-based devices allow researchers to detect unidentified biological markers, which could lead to breakthrough results in the near future. On the other hand, there is still no efficient method for capturing, isolating, enumerating, and characterising CTCs. We are expecting improved CTC capture methodologies in several aspects in the future. The microfluidic device should be reliable and stable in its isolation of CTCs. It should be able to detect as many CTC variants as possible in real-time to meet the clinical demand. In order to fully realise the potential of microfluidics, positive isolation, negative enrichment and highly integrated devices need to be developed to analyse the phenotype and genotype properties of CTCs. A standard operating procedure (SOP) is required for efficiently capturing and isolating CTCs. The design and strategy of capture can vary greatly from device to device. Furthermore, we believe that these novel microfluidic technologies for CTC capture and isolation will be approved by regulatory agencies and used as real-time equipment in the near future.

## Figures and Tables

**Table 1:** Overview of microfluidic devices with CTC isolation mechanism, chip fabrication and other technical parameters.

Isolation Method	Device Fabrication	Device Dimension	Flow Rate	Efficiency	Cancer Cell Lines	Ref.
***Size-based isolation***						
Size and deformability	Double-layer photolithography	L = 500 μm T = 23 μm	2.5 mL/h	~97%	LM2 MDA-MB-231	[93]
Size	Wet etching technique and thermal bonding technique	L = 22 mm H = 40 μm	200 μL/min	85%	BGC823, H1975, PC-3, SKBR3	[82]
Size-based PDMS microflitration membrane	Photolithography	T = 60 μm	10 mL/h	>90%	A549, SK-MES-1, H446	[90]
Size	Photolithography	Main channel L = 80 µm; Main channel L = 50 µm H = 50 µm	10 mL/h	82%	SKBR3, MCF-7, MDAMB231	[105]
***Inertial focusing microchannel-based isolation***						
Label-free, inertial migration of cells	Photolithography	L = 20 mm W = 150 µm H = 50 µm	300 µL/min	>99%	H460, HCC827	[62]
Rotation-induced inertial lift force	photolithography	W = 100, 200, 400 µm D = 30 µm	9 µL/min	90%	U87	[126]
Dean vortex flow, inertial lift force	Photolithography	-	1.7 mL/min	54%	FaDu, CAL27, RPMI2650, UD-SCC9 HNC cells, MDA-MB-468	[120]
Inertial and Dean drag forces	Photolithography	W = 500 μm H = 170 μm	100 μL/min	≥85%	MDA-MB-231, MCF-7, T24	[127]
Inertial microfluidics and Dean flow physics	Photolithography	L = 9.75 mm W = 350 µm	400–2700 μL/min	>94%	MDA-MB-231, Jurkat, K562, HeLa	[128]
Size-dependent lateral migration	Photolithography	Capillary inner and outer diameter = 50 and 360 μm; H = 200 μm L = 5 and 1 cm	200 μL/min	94%	MCF-7	[129]
Self-amplified inertial-focused (SAIF) separation	Photolithography	Zigzag channel W = 40 μm; First expansion region W = 0.84 mm; Second expansion region W = 1.64 mm; H = 50 μm	0.4 mL/min	~80%	A549, MCF-7, HeLa	[121]
Vortex and inertial cell focusing lift force	Photolithography	L = 1000 μm W = 40 μm H = 70 μm; Trapping zone L, W = 720, 230 μm	8 mL/min	83%	MCF-7	[130]
Inertial lift force and Dean drag force	Photolithography	L = 5.5 mm W = 130 μm H = 500 μm	1 mL/min	90%	MCTC	[131]
***Dielectrophoresis-based isolation***						
Optically induced dielectrophoretic (ODEP) force	Metal mould-punching	Main channel, L = 25 mm, W = 1000 μm, H = 100 μm; Side channel, L = 15 mm, W = 400 μm, H = 100 μm	2.5 μL/min	41.5%	PC-3	[135]
Dielectrophoresis at wireless bipolar electrode (BPE) array	Photolithography	L = 2.95 mm W = 200 µm H = 25 µm	20 μm/s	96%	MDA-MB-231, Jurkat E6-1 T	[136]
Dielectrophoresis (DEP) force	Photolithography and wet etching	L = 7 mm H = 50 µm	100 µL/min	92 ± 9%	NCI-H1975	[137]
Optically induced dielectrophoresis (ODEP)	Metal mould-punching	Main channel, L = 2500 µm, W = 1000 μm, H = 60 μm; Side channel, L = 2500 μm, W = 400 μm, H = 60 μm	-	81.0 ± 0.7%	PC-3, SW620	[85]
***Magnetic field-based isolation***						
Immunomagnetics and size-based filtration	Photolithography	T = 50 μm	2 mL/min	~89%	MCF-7	[96]
EpCAM-specific conjugation of MNPs	Photolithography	Microchannel W = 250 μm; Trapping site H = 400 μm, W = 100 μm	150 µL/min	~81.2–96.3%	MDA-MB-231, MCF-7	[89]
EpCAM-based positive method and CD45/CD66b-based negative method by lateral magnetophoresis	Photolithography	Free-bead capture microchannel, L = 42.5 mm, W = 1 mm, H = 50 µm; Lateral magnetophoretic microchannel, L = 42.5 mm, W = 2.8 mm, H = 100 µm	2 mL/h and 3.2 mL/h	83.1%	MDA-MB-231, PC-3, SKBR3, MCF-7	[140]
Magnet deformability	Photolithography	L = 49,000 µm W = 10,000 µm	3 mL/h	90%	HCT116, SW480, MCF-7	[97]
Immunomagnetic technique	Photolithography	L = 9 mm W = 1 mm	-	97–107%	SKBR3, PC-3, Colo205	[99]
Magnetic-ranking cytometry and phenotypic profiling of CTCs	Photolithography	L = 8.75 cm H = 50 µm	500 µL/h	>90%	SKBR3, PC-3, MDA-MB-231	[141]
MNP-labeled aptamers	Photolithography	-	25 mL/h	~79%	PC-3, SKBR3	[142]
Magnetic-bead-mediated dual-antibody functionalised microfluidics	Photolithography	-	0.8 mL/h	>85%	LnCAP and LnCAP-EMP	[143]
Cell size difference in ferrofluids under permanent magnetic influence	Photolithography	L = 2.54 mm W, H = 635 µm	8 µL/min	>99%	HeLa	[144]
Ferrodynamic cell separation	Photolithography	L = 4.94 cm W = 900 µm	6 mL/h	~92.9%	H1299, A549, H3122, PC-3, MCF-7, HCC1806	[145]
***Acoustic-based isolation***						
Cell size difference in ferrofluids	Photolithography	L = 5.81 cm W = 900 µm	20 µL/min	82.2%	A549, H1299, MCF-7, MDA-MB-231	[146]
Lateral cavity acoustic transducers	Photolithography	W = 750 µm H = 100 µm	25 µL/min	94%	Breast, bone, lung cancer cells	[103]
Hydrodynamic and SAW focusing separation	Photolithography	-	7.5 mL/h	>86%	MCF-7, HeLa, PC-3, LNCaP	[150]
Interdigital transducers (IDTs) and focused interdigital transducers (FIDTs) generating standing SAWs and travelling pulsed SAWs	Photolithography	W = 65 µm H = 50 µm	0.3 µL/min	~90%	U87	[151]
Acoustic impedance contrast	Photolithography and deep reactive ion etching (DRIE)	L = 20 mm W = 380 µm H = 200 µm	20–60 µL/min	>86%	HeLa, MDA-MA-231	[152]
Microvortices generated by acoustic vibration	Photolithography	L = 50 mm W = 40 mm H = 200 µm	10 µL/min	>90%	DU145	[153]
Continuous flow acoustophoretic negative selection	Photolithography	Maun channel, L = 20 mm, W = 375 µm, H = 150 µm; Sub channel, L = 10 mm, W = 300 µm, H = 150 µm	100, 400 µL/min	>98%	MCF-7, DU145	[154]
***Combined method-based isolation***						
Inertial and magnetic method	Photolithography	W = 400 µm H = 80 µm	1000 µL/min	~95%	MCF-7	[107]
Vortex trapping and impedance cytometry	-	L = 1 cm H = 70 µm	100 µL/min	~ 98%	MCF-7, LoVo, HT-29 human colon cells,	[95]
Inertial hydrodynamic forces and bifurcation law	CNC micromachining	W = 0.26 mm H = 0.2 mm	-	85%	MCF-7	[155]
Inertial and deformability-based principle	Photolithography	L = 1–1.5 cm W = 400, 300, 200 µm	80 mL/h	>90%	MCF-7	[156]
Integrated device with acoustofluidic label-free separation and direct dielectrophoretic cell trapping	Photolithography	L = 2.3 cm W = 375 µm H = 150 µm	80, 160 µL/min	~76%	DU145	[157]
Inertial-ferrohydrodynamic cell separation	Photolithography	H = 60 µm	~60 mL/h	94.8%	H1299, MDA-MB-231, MCF-7, H3122	[158]
Micropore-arrayed filtration and magnetic bead-functionalised antibody-mediated detection	Molding technique	Micropore L, W = 20 mm, diameter = 10 µm	-	~85%	PC-9	[115]
Lateral cavity acoustic transducers (LCAT) and biomarker-based immuno-labelling	Photolithography	Main, side channel W = 500, 100 µm H = 100 µm	25 µL/min	~100%	MCF-7, SKBR3	[159]
***Electrochemical isolation***						
Antibody-mediated electrochemical release and lysis	Photolithography	L = 40 mm W = 20 mm	1 mL/h	85–100%	PC-3, MCF-7, NCl-H1650	[91]
Electrochemical detection and electric-filed influenced hydrodynamic flow	Screen printing	W = 95 ± 2.5 µm H = 15 ± 1.5 µm	5 µL/min	92 ± 0.5%	HEK-293, HeLa	[116]
***Biological interaction-based isolation***						
EpCAM-expressing cells using antibody-coated microposts	Photolithography	L = 20 mm H = 50–100 µm	1.5–2.5 mL/h	93%	PC-3	[162]
Aptamer-functionalized micropillars	Photolithography	-	1 mL/h	80%	W480 colorectal, LNCap prostate, KATO III gastric cancer cells, K-562 chronic myelogenous leukemia cells	[161]
Anti-EpCAM-coated channel surface with herringbone grooves	Photolithography	L = 50 mm W = 2.1 mm H = 50 µm	1 µL/s	>90%	L3.6pl, BxPC-3, MIAPaCa-2	[163]
EpCAM antibody-functionalised pillars	Laser direct-write technique	Micropost diameter = 420 µm; Pitch = 245 µm	90 µL/min	~76%	HEC-1A	[25]
Combination of anti-EpCAM antibody and anti-N-cadherin antibody	Photolithography	L = 32 mm W = 34 mm H = 0.7 mm	0.6 mL/h	89.6%	SKOV-3 ovarian tumor cells	[104]
Dual aptamer (EpCAM-5-1 and NC3S)-modified poly(lactic-co-glycolic acid) (PLGA) nanofiber	Electrospinning	L = 2 cm W = 1 cm H = 1 mm	300 µL/min	89–91%	A2780, OVCAR-3	[164]
Aptamer-immobilized microchannel	Photolithography	Cell channel W = 1 mm; DNA channel W = 0.5–1 mm H = ~25 µm	5 µL/min	-	HeLa, CAOV-3	[106]
AlGaN/GaN HEMT biosensor array	Photolithography	L = 22 mm W = 13 mm	-	-	HCT-8	[165]
Size-based and multiplex SERS nanovectors	-	Filter gap = 12 µm, H = 40 µm	1 µL/min	~87–93%	SKBR3, MCF7, and MDA-MB-231	[166]
Microchannel functionalised with anti-EpCAM	3D printing	L = 2 cm	1 mL/h	~87–92%	MCF-7, SW480, PC-3, 293T	[81]
Gelatin-coated Ni foam functionalised with anti-WpCAM	Ni foam surface modification	L = 20 mm W = 4 mm H = 1 mm	50 µL/min	~88%	MCF-7	[167]
Lateral displacement (DLD) and herringbone CTC chip functionalised with EpCAM and CD41 antibodies	Deep reactive ion etching	H = 150 µm	1.14 ± 0.24 mL/h	60–83%	Lung, breast, melanoma cancer cells	[168]
EpCAM and CD133 antibodies functionalised hexagonal array of posts	Photolithography	L = 44.6 mm W = 16.9 mm H = 100 µm	1 mL/h	13.6–97.5%	HT-29, Panc-1, PC-3, Hs-578T, Capan-1	[169]
Microcavity array functionalised with anti-EpCAM	Photolithography	H = 200 ± 10 µm Microcavity L, W = 30, 8 µm	0.1 mL/min	~76–83%	MCF-7, SW620	[170]
Magnetic ranking cytometry and CTC surface marker expression	Photolithography	L = 5.4 cm W = 4.3 cm H = 50 µm, Radii of Ni magnet = 145–235 µm	400 µL/h	>90%	LNCaP, PC-3, PC-3M	[171]
Isolation by size of epithelial tumor cell (ISET) and microbeads assisting ISET	-	L = 4 mm W = 17 mm H = 300 µm	1 mL/min	~72–93%	MCF-7, KATO III, PC-3	[172]

## Data Availability

The data that supports this study are available from corresponding authors upon reasonable request.
